# The first complete genome sequence of the African swine fever virus genotype X and serogroup 7 isolated in domestic pigs from the Democratic Republic of Congo

**DOI:** 10.1186/s12985-021-01497-0

**Published:** 2021-01-21

**Authors:** Patrick N. Bisimwa, Juliette R. Ongus, Lucilla Steinaa, Espoir B. Bisimwa, Edwina Bochere, Eunice M. Machuka, Jean-Baka Domelevo Entfellner, Edward Okoth, Roger Pelle

**Affiliations:** 1 Institute of Basic Sciences, Technology and Innovation, Department of Molecular Biology and Biotechnology, Pan African University, Nairobi, Kenya; 2 Department of Animal Science and Production, Université Evangélique en Afrique, P.O. Box 3323, Bukavu, Democratic Republic of Congo; 3 Department of Medical Laboratory Sciences, Jomo Kenyatta University of Agriculture and Technology, Juja, Kenya; 4 Biosciences Eastern and Central Africa-International Livestock Research Institute (BecA-ILRI) Hub, Naivasha Road, P.O. Box 30709, Nairobi, 00100 Kenya; 5 International Livestock Research Institute, Animal and Human Health, Nairobi, Kenya

**Keywords:** African swine fever virus, Complete genome sequence, Initial characterization, Genotype X, South Kivu

## Abstract

**Background:**

African swine fever (ASF), a highly contagious hemorrhagic disease, affects domestic pigs in the Democratic Republic of Congo (DRC) where regular outbreaks are reported leading to high mortality rates approaching 100% in the affected regions. No study on the characteristics of the complete genome of strains responsible for ASF outbreaks in the South Kivu province of DRC is available, limited a better understanding of molecular evolution and spread of this virus within the country. The present study aimed at determining the complete genome sequence of ASFV strains genotype X involved in 2018–2019 ASF disease outbreaks in South Kivu province of DRC.

**Materials and methods:**

Genomic DNA of a spleen sample from an ASFV genotype X-positive domestic pig in Uvira, during the 2018–2019 outbreaks in South Kivu, was sequenced using the Illumina HiSeq X platform. Obtained trimmed reads using Geneious Prime 2020.0.4 were blasted against a pig reference genome then contigs were generated from the unmapped reads enriched in ASFV DNA using Spades implemented in Geneious 2020.0.4. The assembly of the complete genome sequence of ASFV was achieved from the longest overlapping contigs. The new genome was annotated with the genome annotation transfer utility (GATU) software and the CLC Genomics Workbench 8 software was further used to search for any ORFs that failed to be identified by GATU. Subsequent analyses of the newly determined Uvira ASFV genotype X genome were done using BLAST for databases search, CLUSTAL W for multiple sequences alignments and MEGA X for phylogeny.

**Results:**

42 Gbp paired-end reads of 150 bp long were obtained containing about 0.1% of ASFV DNA. The assembled Uvira ASFV genome, termed Uvira B53, was 180,916 bp long that could be assembled in 2 contigs. The Uvira B53genome had a GC content of 38.5%, encoded 168 open reading frames (ORFs) and had 98.8% nucleotide identity with the reference ASFV genotype X Kenya 1950. The phylogenetic relationship with selected representative genomes clustered the Uvira B53 strain together with ASFV genotype X reported to date (Kenya 1950 and Ken05/Tk1). Multiple genome sequences comparison with the two reference ASFV genotype X strains showed that 130 of the 168 ORFs were fully conserved in the Uvira B53. The other 38 ORFs were divergent mainly due to SNPs and indels (deletions and insertions). Most of 46 multigene family (MGF) genes identified were affected by various genetic variations. However, 8 MGF ORFs present in Kenya 1950 and Ken05/Tk1 were absent from the Uvira B53 genome including three members of MGF 360, four of MGF 110 and one of MGF 100 while one MGF ORF (MGF 360-1L) at the left end of the genome was truncated in Uvira B53. Moreover, ORFs DP96R and p285L were also absent in the Uvira B53 genome. In contrast, the ORF MGF 110-5L present in Uvira B53 and Ken05/Tk1 was missing in Kenya 1950. The analysis of the intergenic region between the I73R and I329L genes also revealed sequence variations between the three genotype X strains mainly characterized by a deletion of 69 bp in Uvira B53 and 36 bp in Kenya 1950, compared to Ken05/Tk1. Assessment of the CD2v (EP402R) antigen unveiled the presence of SNPs and indels particularly in the PPPKPY tandem repeat region between selected variants representing the eight serogroups reported to date. Uvira B53 had identical CD2v variable region to the Uganda (KM609361) strain, the only other ASFV serogroup 7 reported to date.

**Conclusion:**

We report the first complete genome sequence of an African swine fever virus (ASFV) p72 genotype X and CD2v serogroup 7, termed Uvira B53. This study provides additional insights on genetic characteristics and evolution of ASFV useful for tracing the geographical spread of ASF and essential for improved design of control and management strategies against ASF.

## Background

African swine fever (ASF), a hemorrhagic contagious viral disease affecting domestic pigs and wild boars, is characterized by high mortality and has become a significant threat to the global pig industry [1]. ASF is caused by African swine fever virus (ASFV), a large DNA virus and the only known member of the genus *Asfivirus* within the *Asfarviridae* virus family [2]. The genome is a double-stranded DNA varying between 170 to 193 kbp in size containing 150 to 167 proteins coding open reading frames (depending on the virus strains) and a conserved central region of about 125 kb, while the ends are variable in size [3]. The epidemiology is complex and embraces various patterns in endemic regions [4, 5]. To date, neither therapy nor vaccine are available against the disease and therefore, the primary prevention methods rely on the application of strict biosecurity measures, early diagnosis and stamping out the infected pig herds [6]. Based on the sequencing of the B646L gene, which encodes the capsid protein p72, 24 genotypes have been currently reported worldwide and all of them are known to circulate in Africa [7, 8].

Previous studies have identified p72 genotype I, IX and XIV to be present in the Democratic Republic of Congo (DRC) [9, 10]. In addition, our recent study has identified for the first time the presence of p72 genotype X in DRC [11] in symptomatic domestic pigs during outbreaks. This emphasize the need for continued characterization of ASFV strains responsible for outbreaks to better understand the spread of the disease in DRC and to develop preventive measures to control the spread. To date, only two complete and fully annotated genomes of ASFV genotype X are available in the GenBank, both of which are from Kenyan virus strains [12]. However, no ASFV complete genome sequencing study has been reported in DRC, an ASFV endemic country, limited a better understanding of molecular evolution and spread of this virus within the country. In this study, we report the first complete genome sequence of ASFV genotype X and serogroup 7 associated with ASF outbreaks in domestic pigs in the South Kivu province in eastern DRC in 2019. Analysis of this ASFV genome in comparison with the previously sequenced and available ASFV genomes provides new insights into genetic diversity of African strains and knowledge that may advance our understanding of host-virus interaction and pathogenicity.

## Materials and methods

### Ethics statement

Ethical approval for the study reported here and the permission for the collection of samples was provided by the Interdisciplinary Centre for Ethical Research (CIRE) established by the Evangelical University in Africa, Bukavu, DRC, with reference (UEA/SGAC/KM 132/2016). A consent form which described the aim of the study was signed by farmers willing to participate in the study after translation into local languages.

### DNA preparation and ASFV detection

A spleen tissue sample was collected during an ASF outbreak from an ASF symptomatic domestic pig in 2019 from the Uvira district of South Kivu province, DRC. The tissue sample was previously confirmed to be infected with ASFV p72 genotype X (11). Genomic DNA was extracted directly from 30 mg of tissue using the DNeasy Blood and Tissue Kit (Qiagen, USA) following the manufacturer’s recommendations. The quality and integrity of the extracted DNA were assessed by 0.8% agarose gel electrophoresis in the presence of 0.5 µg/ml Gel Red (Biotium, Fisher Biotech, Australia) for DNA visualization. The quantity and purity of the DNA were estimated by NanoDrop™ 2000 (Thermo Fisher, USA) spectrophotometer, ensuring that sample with 260/280 OD ratio between 1.8 and 2 were included for downstream analyses. To confirm the presence of ASFV DNA in the sample, polymerase chain reaction (PCR) amplification assay was performed using the ASFV diagnosis primers PPA1/PPA2 (Peste Porcine Africaine) that target the p72 coding region to generate an amplicon of 257 bp [13]. For p72 genotype classification, the C-terminal region of the p72 protein gene (B646L) was amplified and sequenced using primers p72-U/D [5].

### Complete genome sequencing of ASFV

Genohub Inc. (Austin, Texas USA) facilitated the whole genome sequencing service of the genomic DNA purified directly from ASFV-infected pig spleen using the Illumina HiSeq X platform (Illumina, USA). DNA Libraries were prepared using KAPA HyperPlus Kit (KAPA Biosystems, Wilmington, MA) according to the manufacturer’s protocol with 10 ng DNA as input. The final library quality and quantity were analyzed in a Bioanalyzer 2100 (Agilent Technologies, USA) and Qubit™ Fluorometer (Thermo Scientific, USA), respectively. Whole genome sequencing (WGS) of 150 bp paired-end reads were sequenced on Illumina HiSeq X (Illumina Inc., San Diego, CA). A total of 42 Gbp paired-end reads of 150 bp long were obtained.

### Genome assembly and annotation

Cleaning of raw reads was achieved using Geneious Prime 2020.0.4 and a Phred quality score (Q) of 30 and above. The trimmed reads were blasted against the pig reference genome Sus scrofa11.1 from Ensembl (Acc. No. GCA_000003025.6) then the unmapped reads obtained significant hits to ASFV. The original 42 Gbp paired reads contained about 0.1% of ASFV DNA. Contigs were generated using Spades implemented in Geneious 2020.0.4 using default parameters in order to obtain the full genome sequence. Assembly of the complete genome sequence of Uvira B53 was achieved from the longest overlapping contigs. The genome annotation transfer utility (GATU) software at the Viral Bioinformatics Resource Center [14] was used to annotate the newly constructed genome using Ken05/Tk1 ASFV genome as a reference (GenBank Acc. No. NC044945) and CLC Genomics Workbench 8 software (Qiagen, USA) was further used to search for any ORFs that failed to be identified by GATU. A BLAST search was carried out against the NCBI database to confirm the identified ORFs. Moreover, the two ASFV genome sequences were aligned with MAFFT v.7.427 implemented in Geneious software 2020.0.4 to identify substitutions sites. Multiple sequence alignments of retrieved whole genome sequences from the GenBank with Uvira B53 genome were generated using CLUSTAL W [15]. The evolutionary analyses were inferred using unrooted neighbor-joining (NJ) method with MEGA X software [16] and 1000 bootstrap replications were estimated to assess the robustness of individual clades. The same software was used to perform the phylogenetic construction based on the most divergent genes (with several deletions or insertion and with 50–99% sequence identity) using Maximum-likelihood method. The genome sequence generated from this study has been submitted to GenBank under the accession number MT956648.

## Results

### General features of the genome sequence of ASFV Uvira B53 strain

The ASFV Uvira B53 strain is a p72 genotype X isolated from the spleen of an ASFV-positive and symptomatic domestic pig in South Kivu province, East of DRC, during outbreaks reported in the Uvira district in 2019 [11]. Genotype X confirmation of the ASFV Uvira B53 was done through BLAST result showing the highest percentage identity with existing GenBank ASFV sequences of the genotype X (Kenya 1950 GenBank Acc. No. AY261360.1 and Ken05/Tk1 GenBank Acc. No. KM111294.1). The draft assembly of the Uvira B53 genome comprises 180,916 bp that could be reconstructed by assembling the 2 longest contigs (112,709 bp and 68,243 bp), with an average depth of 78.77 reads per base. Therefore, N50, the sequence length in base pairs of the minimum number of contigs to make up 50% of the total genome assembly length, was 112,709 bp (and corresponded to the longest contig), resulting in a L50, the rank of the contig that gave the N50, of 1 (Table 1). Inverted terminal repeats (ITR) were missing at both ends of the genome sequence, probably due to the limited amount of ASFV material (about 0.1% of total reads of 42 Gbp) in the spleen DNA sample sequenced, or because of the complexity of the region and/or difficulties in sequencing/assembly. The base composition of the genome sequence showed a GC content of 38.5%, which is comparable to that of other genotype X strains, i.e. Ken05/Tk1 (38.3%) and Kenya 1950 (38.4%). Sequence annotation using GATU software [14] revealed a total of 168 protein-coding genes (Table 1). In total, 46 multigene family (MGF) genes were identified including MGF 100 (3 members), MGF 110 (13 members), MGF 300 (3 members), MGF 360 (18 members) and MGF 505 (9 members). However, there was a deletion of one MGF 360 member (MGF 360-2L) and 2 other MGF 360 members (MGF 360-21R and MGF 360-1L) in the right variable region; another MGF 360 member in the left variable region (MGF 360-1L) was truncated, being only 95 bp long starting from the 5′ end (compared to the 1071 bp of the complete ORF) and likely non-functional. Also, four MGF 110 members were missing in the Uvira B53 genome (MGF 110-4L, MGF 110-7L, MGF 110-8L and MGF 110-9L) as well as one MGF 100 member (MGF 100-1R).

**Table 1:** Summary of the Uvira B53 ASFV genomic sequencing data

Genome assembly	Number of contigs	Largest contig	Total length (bp)	% GC content	N50 (bp)	L50	ORFs
ASFV Uvira B53	9	112,709	180,916	38.5	112,709	1	168

### Initial comparative analysis of the Uvira B53 strain genome with other ASFV strains

A total of seventeen ASFV complete genome sequences representing different strains reported in several ASF endemic countries were retrieved from the GenBank and were used in this study for comparison and phylogeny. Genome’s GenBank accession numbers, country and year of isolation, virus genotype, host, global alignment percentage identity to the genome of ASFV strain Uvira B53, and the reference are shown in Table 2. The pair-wise alignment between the Uvira B53 strain and other ASFV genomes showed the highest maximum percentage identity with strains of the genotype X, specifically Kenya 1950 (98.85%) and Ken05/Tk1 (95.52%) (Table 2)*.*

**Table 2:** Comparison of complete genome sequences of Uvira B53 ASFV with selected genomes from the GenBank

Strain name	GenBank Acc. No	Country	Year	p72 genotype	Host	% identity to Uvira B53	Reference
Tengani 62	AY261364	Malawi	1962	V/I	Pig	86.6	Pan [17]
Georgia 2007/1	FR682468	Georgia	2007	II	Pig	85.4	Chapman et al. [18]
Kenya 1950	AY261360	Kenya	1950	X	Pig	98.8	Zsak et al. [19]
Kenya05/Tk1	NC044945	Kenya	2005	X	Tick	95.3	Bishop et al. (2015)
Benin 97/1	NC044956	Benin	1997	I	Pig	86.2	Chapman et al. (2008)
Ken06.Bus	KM111295	Kenya	2006	IX	Pig	92.2	Bishop et al. [20]
R35	MH025920	Uganda	2015	IX	Pig	91.9	Unpublished
N10	MH025919	Uganda	2015	IX	Pig	91.9	Unpublished
Pretorisuskop/96/4	AY261363	South Africa	1996	XX/I	Tick	86.1	Zsak et al. [19]
Warthog	AY261366	Namibia	1980	IV	Warthog	86.5	Zsak et al. [19]
R8	MH025916	Uganda	2015	IX	Pig	91.9	Unpublished
Mkuzi 1979	NC044953	South Africa	1979	I/VII	Tick	85.7	Zsak et al. [19]
Belgium 2018/1	LR536725	Belgium	2018	II	Wild boar	85.4	Pikalo et al. [22]
RSA_2_2004	MN641877	South Africa	2004	XX	Wild boar	84.6	Unpublished
Zaire	MN630494	Zaire	2020	XX	Pig	85.4	Unpublished
Pig/China/Cas19-01/2019	MN172368	China	2019	II	Pig	85.4	Jia et al. [23]
85/Ca/1985	MN270973	Italy	1985	I	Pig	86.3	Torresi et al. [24]

### Genome comparison of ASFV Uvira B53 with reported ASFV genotype X strains

**Fig. 1:**
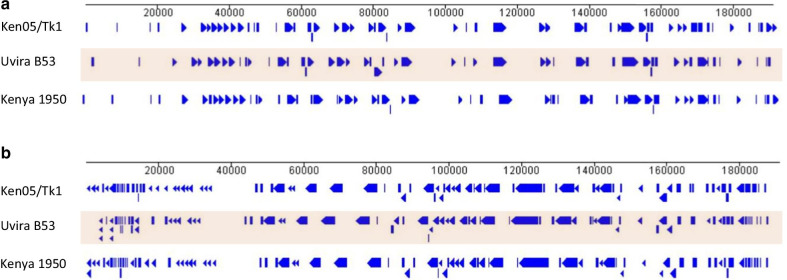
Linear genomic profiles of Uvira B53 (shadowed) and related ASFV genotype X strains. Positions of protein-coding genes are indicated by plain arrows and bars, with the direction of the arrows indicating the 5′ to 3′orientation of the coding sequence. Panel A represent homologous genes on the sense (forward) strand and panel B are homologous genes on the antisense (reverse) strand. Numbers below the lines indicate bp. The map was generated using the Viral Orthologous Cluster V.2.0 software for *Asfarviridae* implemented in the Bioinformatics Centre

Comparatively, with a length of 180,916 bp, the newly determined Uvira B53 genome is about 10–13 kbp shorter than the two reference genotype X strains from the GenBank, i.e. Kenya 1950 (193,886 bp) and Ken05/Tk1 (191,058 bp). However, Uvira B53 was genetically closer to Kenya 1950, a pig-derived strain exhibiting 98.8% DNA identity, than to Ken05/Tk1, a tick-derived strain with 95.3% identity (Table 2). A visual representation of the whole genome alignment of homologous genes between the ASFV genotype X generated using the Viral Orthologous Cluster V.2.0 [14] is shown in Fig. 1. Sequence alignment showed that the length difference observed between these genomes is due mainly to the absence of some genes in Uvira B53 particularly the members of the 5 multigene families such as MGF 100, MGF 110, MGF 300, MGF 360, and MGF 505. In summary, 10 genes were not present in the Uvira B53 genome while present in the two Kenyan genotype X including 8 multigene families (MGF 360-2L, MGF 110-4L, MGF 110-7L, MGF 110-8L, MGF 100-1R, MGF 110-9L, MGF 360-21R and MGF 360-1L), DP96R encoding the UK protein [25] and p285L of unknown functions (Table 3). More specifically, MGF 360-1L and MGF 360-21R were absent in the right terminal in the Uvira B53 strain while they were present in the other genotype X strains. Moreover, MGF 110-4L, MGF 110-7L, MGF 110-8L, MGF 110-9L and MGF 100-1R were absent in Uvira B53 strain but present in the two reference genotype X strains. In contrast, MGF-110-5L was absent in the Kenya 1950 isolate but present in the Uvira B53 and Ken05/Tk1 (Table 3).

**Table 3:** ORFs present in Kenya 1950 and Ken05/Tk1 but absent in Uvira B53

ORF name	Uvira B53	Kenya 1950	Ken05/Tk1
DP96R	–	+	+
MGF 100-1R	–	+	+
MGF 110-4L	–	+	+
MGF 110-7L	–	+	+
MGF 110-8L	–	+	+
MGF 110-9L	–	+	+
MGF 360-1L (right)	–	+	+
MGF 360-21R (right)	–	+	+
MGF 360-2L	–	+	+
p285L	–	+	+
MGF 110-5L^a^	+	–	+

Abbreviation: ( +), ORF present; (-) ORF not present

^a^MGF 110-5L is present in Uvira B53 but absent in Kenya 1950

Overall, of the 168 ORFs identified in the Uvira B53 genome, 134 shared 100% identity with homologs in Ken05/Tk1 whereas the 34 others were polymorphic (56.6 to 99.6% identity). On the other hand, Uvira B53 and Kenya 1950 also shared 167 ORFs including 136 with 100% sequence identity and 31 that were divergent (74.4% to 99.7% sequence identity). Therefore, one Uvira B53 ORFs (MGF 110-5L) was absent in Kenya 1950. Altogether, the 168 ORFs in the Uvira B53 genome could be clustered into two main groups: 130 conserved and 38 non-conserved ORFs (Table 4).

**Table 4:** Uvira B53 polymorphic ORFs with respect to Ken05/Tk1 and Kenya 1950

Gene	Function	References	% sequence identity to	
			Ken05/Tk1	Kenya 1950
A859L	Helicase superfamily II	[14]	99.4	100
B117L	Transmembrane region containing protein	[14]	90	77.1
B169L	Putative signal peptide	[14]	91.5	98.8
B407L	Unknown	[14]	97.3	99.5
B475L	Unknown	[14]	99.6	95.8
C84L	Putative signal peptide	[14]	98.5	97
CP123L	Putative signal peptide	[14]	100	99.2
D129L	Unknown	[14]	86	92.7
E146L (j16L)	Putative signal peptide	[14]	98.6	100
E183L (p54, j13L)	Structural protein p54	[14]	98.4	91.4
EP153R	Lectin-like protein	[14]	84.3	79.5
EP364R	Predicted nuclease and potential DEATH domain	[14]	95.5	97.1
EP402R (CD2v)	CD2-like protein	[14]	76	96.2
I10L	Unknown	[14]	100	97.6
I10L_2	Unknown	[14]	61.8	96
I12R	Unknown	[14]	88.3	78.7
I177L (k14L)	Putative signaling peptide	[14]	90.1	100
I196L (k15L)	Putative signaling peptide	[14]	79.4	94.9
I8L	Unknown	[14]	62.1	100
I9R	Unknown	[14]	97.9	99
KP177R	Structural protein p22	[14]	90.9	98.8
L60L	Unknown	[14]	52.1	74.4
O61R (p12)	Structural protein p12	[14]	98.4	96.8
QP383R (j11R)	NifS-like PLP-dependent transferase	[14]	90.3	99.7
X69R	Putative signal peptide	[14]	95.8	84.9
MGF 110-11L	Unknown	[14]	76.1	92.1
MGF 110-13L-14L	Unknown	[14]	96.7	95.3
MGF 110-2L	Unknown	[14]	83.7	95.6
MGF 110-5L	Unknown	[14]	98.4	(-)
MGF 110-6L	Unknown	[14]	65.3	95.1
MGF 300-2R	Unknown	[14]	50.6	100
MGF 300-4L	Unknown	[14]	54.4	96.4
MGF 360-13L	Virus replication, type I IFN Inhibitors	[3, 26]	74.8	99.2
MGF 360-18R	Virus replication, type I IFN Inhibitors	[3, 26]	82.5	97.7
MGF 360-6L	Virus replication, type I IFN Inhibitors	[3, 26]	85.5	94.9
MGF 360-8L	Virus replication, type I IFN Inhibitors	[3, 26]	78.7	91.1
MGF 505-1R	Virus replication, type I IFN Inhibitors	[3, 26]	72.5	98.1
MGF 505-4R	Virus replication, type I IFN Inhibitors	[3, 26]	100	99.2

**(–),** ORF not present

### Conserved ORFs

The conserved category included 130 Uvira B53 ORFs for proteins showing 100% amino acid identity with the two reference ASFV genotype X analyzed. Some of them encode for structural proteins, transcription, replication and processing factors, enzymes and proteins involved in nucleotide metabolism, and DNA repair. Whereas several other ORFs were classified as coding for membranes proteins from which 16 belong to the members of MGF. Also clustered in the category of conserved ORFs were protein-coding genes A238L (an IkB-like protein), H339R (the viral protein involved in host-virus interaction), E301R (proliferating cell nuclear antigen), B263R (the TATA box binding protein), Bcl-2 A179L (the apoptosis regulating protein) and E120R (the DNA-binding structure). Furthermore, most conserved proteins included several uncharacterized ORFs such as F317L, H171R (with 100% identity between the strains) (Data not shown).

### Non-conserved, variable ORFs

Sequence comparison revealed that 38 Uvira B53 ORFs were polymorphic in either Ken05/Tk1 or Kenya 1950, or both strains, with 50.6–99.6% sequence identity (Table 4). In comparison with Ken05/Tk1, non-conserved ORFs included six proteins which contain the putative signal peptide and transmembrane region (B169L, C84L, E146L, I177L, I196L, and X69R), one belonging to helicase superfamily II (A859L), the structural protein p54 (E183L and KP177R), the lectin-like protein (EP153R), the CD2 homolog (EP402R), a ERCC4 predicted nuclease and potential death domain (EP364R), NifS-like PLP-dependent transferase (QP383R), twelve members of the MGF including five of MGF 110, (2L, 5L, 6L, 11L, and 13L), two MGF 300 (2R and 4L), four MGF 360 (6L, 8L, 13L and 18R), one MGF 505 (1R) and eight ORFs of unknown functions.

With respect to Kenya 1950 strain, we identified 31 ORFs with 74.4% to 99.7% sequence identity (Table 4). One ORF (MGF 110-5L) was missing in Kenya 1950 while present in the other strains.

### Comparison of the region between I73R and I329L genes in the Uvira B53 and other genotype X strains

**Fig. 2:**
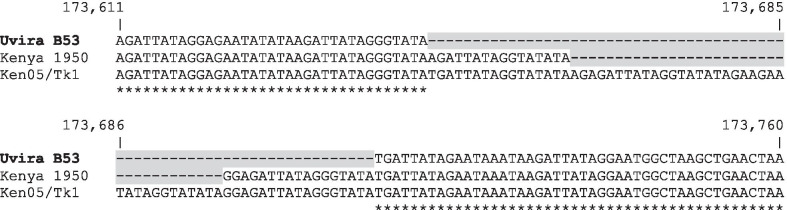
Partial nucleotide sequence alignments of the intergenic region between *I73R* and *I329L* genes of African swine fever virus (ASFV) genotype X strains. The intergenic region ranging from nucleotides 173,611 to 173,760 presents sequence size variations due to indels (colored in yellow): compared to Ken05/Tk1, Uvira B53 contains a 69 bp deletion whereas Kenya 1950 has a 36 bp deletion. Nucleotide residues conserved in all sequences are identified below the alignment (*)

Previous studies have demonstrated that ASFV genotype X strains reported to date are closely related and are known to be widespread in Kenya [20]. Some small length variations among these strains’ genomes are mostly due to the number of tandem repeat sequences (TRS) either within genes or within intergenic regions. The intergenic region between I173R and I329L genes is essential for discriminating between closely related ASFV strains. In that regard, we assessed the tandem repeat sequence in the intergenic region between those two genes (region 173,611–173,760) in the Uvira B53 and the two Kenyan genotype X strains. The multiple sequence alignment revealed a significant size variation due to indels. With respect to Ken05/Tk1, Uvira B53 showed a 69 bp deletion, whereas Kenya 1950 featured a 36 bp deletion (Fig. 2).

### Phylogenetic analysis of the complete genomes of ASFV strains and the polymorphic genes

**Fig. 3:**
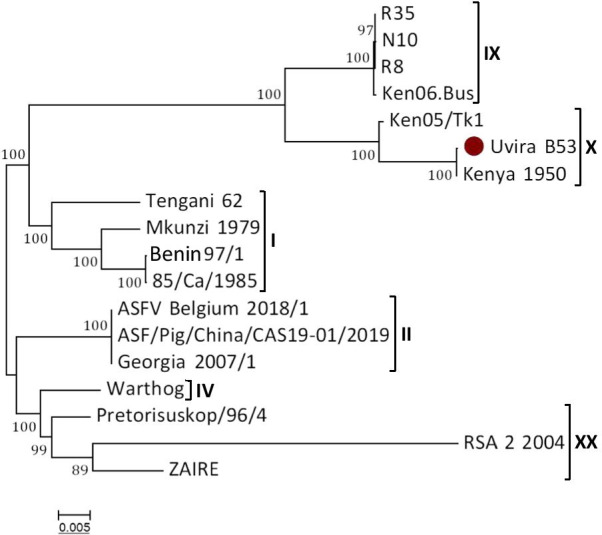
Neighbor-Joining phylogenetic unrooted tree constructed from multiple nucleotide sequence alignments of genomes of 18 ASFV strains. The scale bar is given in numbers of substitutions per site. Phylogeny was inferred following 1,000 bootstrap replications, and the node values show percentage bootstrap support. The genotype status of analyzed viral genomes is indicated. Sequence from this study is presented by plain circle (•)

The genetic relationship between the ASFV strains was assessed through multiple sequence alignments of the whole complete genome sequences from 17 representative ASFV strains retrieved from the GenBank. Phylogenetic analysis grouped the viruses into different clusters corresponding to their genotypes as expected. Thus, Uvira B53 clustered with the two other ASFV p72 genotype X, Kenya 1950 and Ken05/Tk1 strains (Fig. 3). The closest but distinct cluster to this genotype X group was the cluster composed of genotype IX ASFV strains (Ken06.Bus, R8, R35 and N10) whereas the most distantly related clusters concerning Uvira B53 included genotype XX with virus strains from DRC (Zaire) and South Africa (Pretorisuskop/96 and RSA_2_2004), genotype II containing the Georgia, Belgium and China strains as well as genotype IV containing the Namibian warthog strain.

**Fig. 4:**
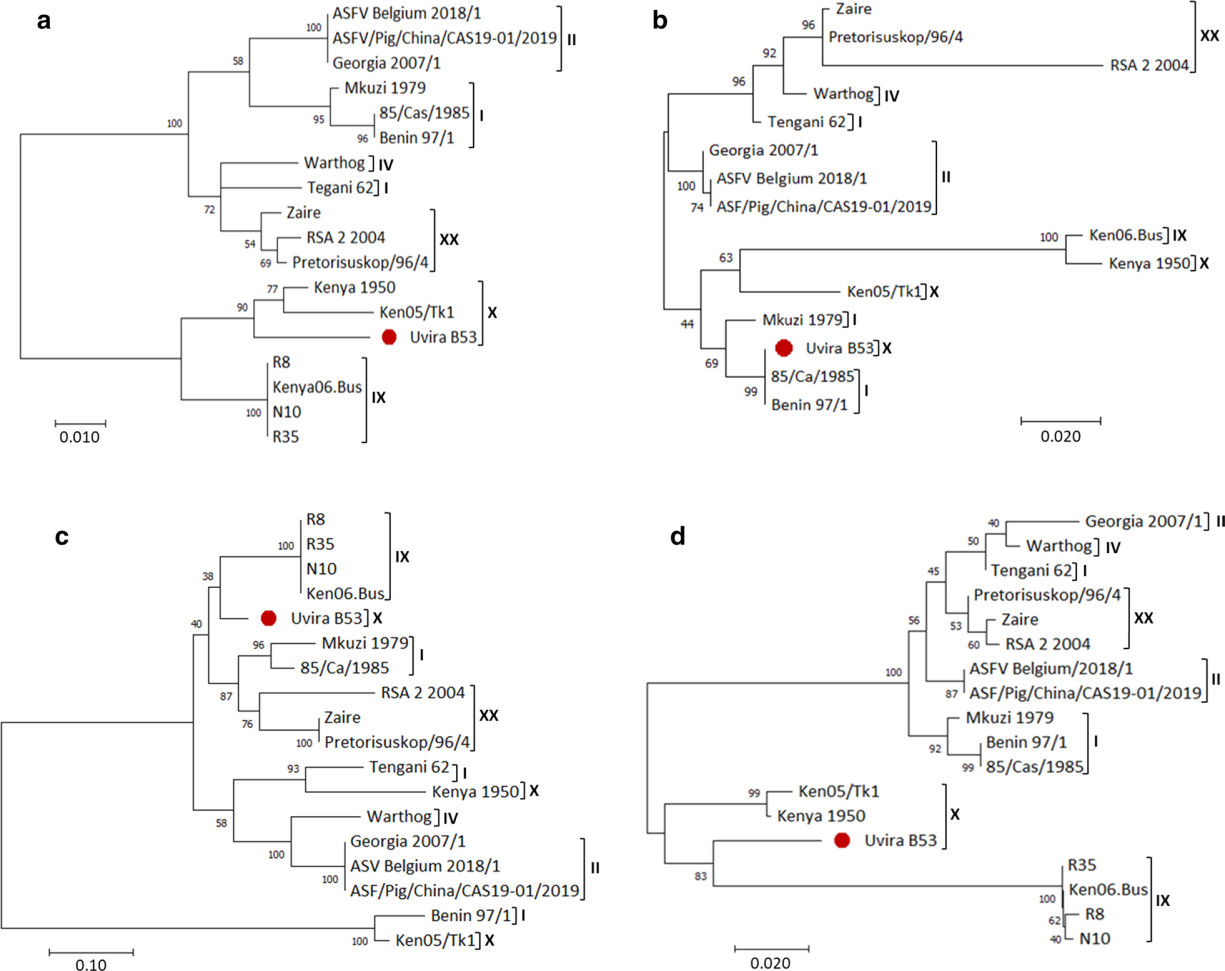
Phylogenetic comparison of gene products of the four most divergent ORFs across 18 African swine fever virus strains. Putative signaling peptide I196L (**a**), the structural protein p22 KP177R (**b**), lectin-like EP153R (**c**) and putative signaling peptide I177L (d). Uvira B53 gene products are presented by plain circle (•)

Furthermore, we looked at polymorphic genes among all the 18 ASFV strains and carried out a phylogenetic analysis of four of these highly divergent genes, which included I196L, KP177R, EP153R and I177L. The Uvira B53 protein variants for I196L and I177L genes clustered with the Kenyan strains Kenya 1950 and Ken05/Tk1 from the 15 other strains analyzed (Fig. 4a, d). In contrast, protein variants encoded by the two other genes, KP177R and EP153R, showed hypervariable regions among the strains and separated the three ASFV genotype X into different clusters (Fig. 4b, c). The KP177R gene product clustered Uvira B53 (genotype X) together with Benin 97/1 and 85/Ca/1985 (genotype I). The KP177R gene was absent in the Ugandan strains (R8, R35 and N10) genotype IX. In contrast, given the low bootstrap percentage (38%) of the node value, the EP153R protein grouped Uvira B53 with Ugandan strains (R8, R35, N10) and Kenyan strain Ken06.Bus (Fig. 4c).

### Amino acid sequence comparison of the EP402R (CD2V) and serotyping

To determine the hemadsorption inhibition (HAI) and serogroup characteristics, the protein sequence of Uvira B53 EP402R gene was compared with the ones of 18 other ASFV strains retrieved from the GenBank and of which 13 representing the 8 serogroups known to date. The results revealed a high sequence variation in the CD2v protein among all the strains. The Uganda (KM609361) strain of serogroup 7 was the most closely related to Uvira B53 displaying 99% amino acid identity (the Uvira B53 CD2v is 373 amino acid long, three amino acid residues longer than its Uganda counterpart; data not shown), suggesting that Uvira B53 reported in this study belongs to serogroup 7, representing the second ASFV serogroup 7 reported to date.

**Fig. 5:**
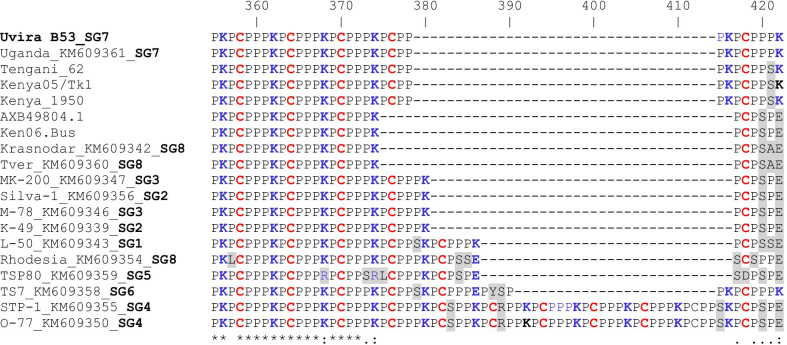
Amino acid sequence of partial EP420R gene products of Uvira B53 strain compared with 18 selected reference strains showing the tandem repeats. Amino acid variations and deletions between strains are shown with shadow and dashes, respectively. Names of the strains are followed by the GenBank accession numbers and serogroup (SG) where known. The conserved amino acid residues in all sequences are identified below the alignment by an asterisk (*)

The C-terminal end of CD2v is characterized by a tandem repeat sequence (TRS) of six amino acids PPPKPC. Comparative analysis of the partial TRS region showed sequence diversity due to amino acid substitutions and deletions (Fig. 5). However, O-77 and STP-1 strains, both of serogroup 4, did not contain indels in these partial sequence alignments thus displaced the longest TRS. For Uvira B53 and Uganda (KM609361) strains, this partial TRS was identical.

## Discussion

In this study, we generated the first complete genome sequence of an ASFV strain, the Uvira B53, directly from a spleen tissue sample of ASF symptomatic domestic pig sourced from Uvira district in South Kivu province, eastern DRC in 2019. Overall, the assembled 180,916 bp long genome sequence obtained was of a high-quality BLAST-match with the previously reported p72 genotype X sequences available in the GenBank. The Uvira B53 ASFV strain is genetically more closely related to the Kenyan strains genotype X than to other ASF viruses of different genotypes.

Compared to the two published genotype X which are all from Kenya, the Uvira B53 genome sequence contained a group of fully conserved genes and a group of divergent genes with SNPs and/or indels (insertions/deletions). In addition, a couple of ORFs found in the two Kenyan genotype X were absent in the Uvira B53 strain: DP96R coding for the UK protein [25] and p285L with an unknown function. The absence of the DP96R gene has also been reported in the virulent Ken06.Bus strain genotype IX [20] and the p285L gene was reported in the virulent Benin 97/1 strain [21]. DP96R is among the known ASFV virulence factors [27]. Based on this information, the absence of DP96R in Uvira B53 may suggest that the virulence of the DRC strain is different from that of the Kenyan strains genotype X. However, DP96R and p285L were found in the non-virulent strain OURT 88/3 [21] as well as in the Malawian virulent strain Tengani 62 [17]. Therefore, further study and information are needed to determine the role of these genes in Uvira B53 and improve our understanding of variable levels of pathogenicity between different ASFV strains.

Like in the two published genotype X, Kenya 1950 and Ken05/Tk1, the terminal inverted repeats were missing at both ends of the Uvira B53 genome sequence. This could be due to lack of enough ASFV DNA in the spleen DNA sample sequenced as we did not use any virus enrichment method (e.g. filtration, cell culture or ultracentrifugation) during viral DNA preparation; furthermore, it could also be due to the difficulty to sequence extensive homopolymer and ITR regions of the ASF virus genome. In addition, the Illumina sequencing platform used generates only short reads, which makes it difficult to effectively sequence and assemble repetitive regions as short repeats could collapse. The ASFV genome sequence represented 0.1% of the 42 Gbp total reads obtained, which corresponded to 42 Mbp of virus sequences. Assuming that the ASFV genome was about 200 kbp long, then the 42 Mbp reads will correspond to 210 × genome coverage. In support to this estimation, our two longest contigs could assemble into the 180,916 bp long genome with an average depth of 78.77 reads per nucleotide. Therefore, the amount of virus DNA used should not be the main cause of the observed sequencing limitation but rather the short length of reads, which could be addressed by using a sequencing platform that will generate long reads.

Several non-conserved divergent genes were identified to be affected by mutations including three proteins involved in putative signal peptide and transmembrane region (C262R, CP123L and E248R), one helicase superfamily II (F1055L), one AP endonuclease class II (E296R) and one RNA polymerase subunit 3 (H359L). However, the effects of these substitutions on protein function are unknown.

Several genetic variations including SNPs, indels (insertions/deletions) and complete loss of ORFs were observed within the MGF member genes between Uvira B53 and known genotype X strains, specifically in genes previously reported to be implicated in determining host range and virulence such as MGF 360, MGF 505 and MGF 110. Indels and loss of ORFs were among the major causes of the differences in length between these genomes as previously reported [28]. They were observed in all the three strains genotype X analyzed but at higher magnitude in Uvira B53 than the two other strains, making the newly completed 180,916 bp long Uvira B53 genome 10 and 13 kbp shorter than the Kenya 1950 and Ken05/Tk1 genomes, respectively. The biological implications of these variations remain to be determined.

The high level of genetic conservation was observed in some ORFs of MGF 360 (9L, 10L, 13L, 15R and 16) and MGF 505 (3R, 4R and 10R) among Uvira B53 and Kenya 1950, a virulent strain [20] whereas they were absent in the Ken05/Tk1, an avirulent strain [20]. This feature may suggest difference and similarity in the Uvira B53 pathogenicity with Ken05/Tk1 and Kenya 1950, respectively. This hypothesis is in agreement with a previous study that demonstrated that some members of MGF 360 and MGF 505 such as MGF360-12L, -13L, and -14L and MGF505-1R, -2R, and -3R were associated with ASFV host range specificity, blocking of the host innate response, and virus virulence, as they were present in several virulent strains of ASFV while absent in the avirulent strains like OURT 88/3 and NHV [29–31]. Further analysis of these differences should provide more insights into the molecular mechanisms underlying the virulence of Uvira B53 genotype X in pigs in South Kivu.

The comparative study based of the intergenic region between I73R and I329L genes showed the genome sequence of the Uvira B53 strain was closer to Kenya 1950 than Ken05/Tk1, as both the Uvira B53 and Kenya 1950 contained deletions compared to Ken05/Tk1. However, the Uvira B53 deletion was 33 bp longer than that of Kenya 1950. This observation corroborates our previous comparative analysis of this intergenic region which revealed this deletion in genotype X strains from symptomatic pigs in South Kivu, DRC [11].

Analysis of phylogenetic relationship was performed using some representative sequence data sets retrieved from the GenBank. The analysis clustered the ASFV Uvira B53 together with genotype X strains reported in Kenya. This result is in accordance with our previous study reporting circulation of ASFV genotype X in symptomatic ASFV infected pigs from South Kivu province [11]. Uvira B53 is different from genotype IX, I and XIV that have recently been reported in some provinces of DRC such as Bandundu, Equateur, Katanga, Kinshasa, Maniema and Province Orientale [10]. They are sylvatic cycle-associated genotype that has been identified from ticks, warthog and domestic pigs in Kenya, Tanzania and Burundi [5, 32–34]. The phylogenetic analysis of some of the divergent protein-coding gene products, particularly the I196L and I177L, matched with the phylogenetic relationship of the complete genome sequence that grouped the Uvira B53 with Kenya 1950 and Ken05/Tk1 genotype X strains. Two other gene products (EP153R and KP177R) were highly divergent in all the strains. A similar observation was reported by Chapman et al. [18] where phylogenetic analyses of several divergent gene products did not match with one of the complete genome sequences. The divergence may probably be due to the occurrence of recombination events and antigenic diversity. Because of this, we suggest to be cautious when performing phylogenetic relationships between ASFV strains based on a small number of genes.

Several variations were identified in the C-type lectin-like protein (EP153R) in all the strains, making it one of the most divergent genes. Variations in this protein may alter the expression of the functional protein (C-type lectin) that may influence the immuno-modulatory functions as previously demonstrated [34].

In addition to SNPs, variations in this region of CD2v (EP402R) were characterized by the presence of the tandem repeat amino acid sequence PPPKPY [35, 37, 38] that was repeated 3–11 times among the strains analyzed. This CD2v variable region is identical in the Uganda (KM609361) strain of serogroup 7 and Uvira B53 with 5 tandem repeats. BLAST search also revealed that the Uganda (KM609361) strain’s CD2v antigen was the most closely related antigen to Uvira B53′s CD2v, displaying 99% amino acid identity. This finding implied that Uvira B53 strain belongs to the ASFV serogroup 7. However, Kenya 1950 found to be the closest genotype to Uvira B53 was shown to cluster with ASFV serogroup 6 based on C-type lectin protein sequence (35). Our study is the first report of a complete genome sequence of ASFV of the serogroup 7. In addition to the serogroups 1 and 2 already reported in DRC [36], ASFV Uvira B53 genotype X and serogroup 7 represents the third ASFV serogroup to be found in DRC.

## Conclusion

African swine fever outbreaks were reported in South Kivu in 2019 causing mortality in domestic pigs. We report for the first time the complete genome sequence of the ASFV Uvira B53, a genotype X and serogroup 7 strain involved in outbreaks in South Kivu. Its similarity with Kenya 1950 strain based on MGF members suggests that it is a virulent strain, corroborated by the symptomatic phenotype of the pig from which the sequenced strain originated. Uvira B53 is the third ASFV genotype X genome sequence, after Kenyan ones, reported to date. The uncontrolled transboundary movements of pigs and pig products in the region together with our data and previous reports on the presence of ASFV p72 genotype X (10, 12, 38, 39) raise the point that Uvira B53 could derive from Kenya 1950. However, further phylogenomic analysis is required to ascertain this relationship. The new genomic information reported in this study provides further insights for tracing geographical spread and biological evolution of the ASF virus, which are essential for the identification of origin and chains of transmission of the pathogen as well as the design of prevention and control strategies of this disease.

## Data Availability

The datasets used and/or analyzed during the current study are available from the corresponding author on reasonable request.

## Abbreviations

ASF: African swine fever
ASFV: African swine fever virus
BLAST: Basic local alignment search tool
DRC: Democratic Republic of Congo
DNA: Deoxyribonucleic acid
GATU: The genome annotation transfer utility
Kbp: Kilo base pair
MGF: Multigene family
NCBI: National Center for Biotechnology Information
ORF: Open reading frame
PAUISTI: Pan African University Institute of Science Technology and Innovation
TRS: Tandem repeat sequence
UEA: Université Evangélique en Afrique
